# Plasmonic Sensing Studies of a Gas-Phase Cystic Fibrosis Marker in Moisture Laden Air

**DOI:** 10.3390/s21113776

**Published:** 2021-05-29

**Authors:** Libin Sun, Douglas Conrad, Drew A. Hall, Kurt D. Benkstein, Steve Semancik, Mona E. Zaghloul

**Affiliations:** 1 School of Engineering and Applied Science, George Washington University, Washington, DC 20052, USA; 2 Biomolecular Measurement Division, National Institute of Standards and Technology, Gaithersburg, MD 20899, USA; kurt.benkstein@nist.gov (K.D.B.); stephen.semancik@nist.gov (S.S.); 3 Department of Medicine, University of California, San Diego, CA 92037, USA; dconrad@health.ucsd.edu; 4 Department of Electrical and Computer Engineering, Jacobs School of Engineering, University of California, San Diego, CA 92093, USA; drewhall@ucsd.edu

**Keywords:** localized surface plasmon resonance, plasmonic sensing, image processing, cystic fibrosis, acetaldehyde, humidity

## Abstract

A plasmonic sensing platform was developed as a noninvasive method to monitor gas-phase biomarkers related to cystic fibrosis (CF). The nanohole array (NHA) sensing platform is based on localized surface plasmon resonance (LSPR) and offers a rapid data acquisition capability. Among the numerous gas-phase biomarkers that can be used to assess the lung health of CF patients, acetaldehyde was selected for this investigation. Previous research with diverse types of sensing platforms, with materials ranging from metal oxides to 2-D materials, detected gas-phase acetaldehyde with the lowest detection limit at the µmol/mol (parts-per-million (ppm)) level. In contrast, this work presents a plasmonic sensing platform that can approach the nmol/mol (parts-per-billion (ppb)) level, which covers the required concentration range needed to monitor the status of lung infection and find pulmonary exacerbations. During the experimental measurements made by a spectrometer and by a smartphone, the sensing examination was initially performed in a dry air background and then with high relative humidity (RH) as an interferent, which is relevant to exhaled breath. At a room temperature of 23.1 °C, the lowest detection limit for the investigated plasmonic sensing platform under dry air and 72% RH conditions are 250 nmol/mol (ppb) and 1000 nmol/mol (ppb), respectively.

## 1. Introduction

Nearly 70,000 individuals worldwide suffer from cystic fibrosis, a systemic genetic disorder induced by mutations in the Cystic Fibrosis Transmembrane Conductance Regulator (CFTR) gene [1,2,3,4]. Mutations of the CFTR gene cause mucus dysfunction, leading to polymicrobial airway infections, which intermittently exacerbate symptoms and result in rapid lung function loss [4,5,6]. Although significant advancements in CFTR gene modulator therapy and other therapeutic approaches have improved medical outcomes, the disease is not considered cured and is still associated with significant morbidity and mortality [2,3,5,7]. Current methods used by physicians to monitor the respiratory health of CF patients, such as spirometry, require specialized equipment and trained personnel [2,8,9]. Hence, there is an unmet need for monitoring approaches that are quick, inexpensive, and require little equipment and patient training. Such technology could warn of pulmonary deterioration before it is necessary to hospitalize or administer intravenous antibiotics to the patient.

Considerable research has focused on biomarkers in exhaled breath condensate (EBC) or sputum, targeting the detection of pulmonary illness [9]. In contrast, this work aims to directly track an exhaled gas-phase biomarker related to cystic fibrosis, specifically acetaldehyde (CH_3_CHO). Real-time breath analysis presents many potential benefits. As such, there are various technologies such as electrochemistry [10], gas chromatography (GC) [11], mass spectrometry (MS) [11,12], and optical spectroscopy [13,14] that have been investigated and published over the last few decades. Currently, the conventional laboratory-based techniques for analyzing volatile organic compounds (VOCs) are thermal desorption (TD) GC-MS and nuclear-magnetic-resonance (NMR) [15,16,17]. These techniques require trained technicians, occupy considerable space, and are expensive, precluding their use as point-of-care (POC) devices. To address these issues, the proposed sensing platform in these studies is a potential technology that might allow self-monitoring of the chronic CF airway infection.

This paper describes a plasmonic sensing platform that provides an opportunity to assess the microbial community’s metabolism in the airways of CF patients. The symptoms associated with the pulmonary exacerbations in CF patients are associated with changes in microbial community metabolism from respiration (anaerobic and aerobic) to fermentation. This transition to fermentation occurs when mucus accumulation results in diminished oxygen concentration in airway mucus plugs. Published literature has revealed that the reduced oxygen concentration encourages the fermentation and growth of anaerobic bacteria, as well as the generation of fermentative metabolites, such as acetaldehyde, ethanol, and 2,3-butanediol [4,15,16]. Previous studies have shown that exhaled breath of CF patients contains up to about 1120 nmol/mol of acetaldehyde, which is almost an order of magnitude higher than the acetaldehyde in a healthy individual’s breath [13,17]. This physiologically relevant concentration range (from 140 nmol/mol to 1120 nmol/mol) is covered by the sensing platform presented in this paper. The sensing platform consists of a nanohole array (NHA), which is coated with a copper benzenetricarboxylate (Cu-BTC) metal-organic framework (MOF). Our previous work has indicated that such NHAs with a Cu-BTC MOF layer can detect VOCs at nmol/mol levels [18,19,20]. When target gas molecules adsorb in the MOF layer, peak wavelength shifts and intensity changes of localized surface plasmon resonance (LSPR) features can be monitored by a spectrometer. Initial measurements also indicate that, with further development, this platform can also be utilized as a portable POC device by substituting a CMOS (complementary metal-oxide-semiconductor) imaging device for the spectrometer and employing artificial neural networks (ANN) for analysis [21]. To further increase the platform portability, it is possible to replace the imager with a smartphone camera. On account of the widespread popularity of smartphones, patients will be able to use their own smartphones with the proposed POC device to complete biomarker detection, which further reduces costs. In addition, using a smartphone can offer benefits from its processor, which allows patients to complete image processing, analysis, and display after acquisition with suitable software.

## 2. Detection Principle

The plasmonic sensing platform features subwavelength NHA structures designed for incident light in the visible spectrum range to result in LSPR phenomena. The NHA structure effectively couples the incident light wave (electromagnetic radiation) with surface plasmons (SPs) that are sensitively affected by the local environment [20,22]. The nanoholes’ dimension (200 nm) is much smaller than the wavelength of the incident light, allowing the conduction electrons inside the gold layer of the NHAs to polarize and accumulate on the surface of the NHAs. These polarized SPs at the metal and dielectric layer interface couple with the incident light, and resonant oscillations occur at specific frequencies [22,23]. This leads to a local enhancement of the electromagnetic field in proximity to the NHA area that improves the performance of the plasmonic sensing platform. Environmental changes occurring in the vicinity of features in the NHA can also cause measurable changes to the LSPR characteristics [18,19,22,23]. When performing response studies, gas molecules are adsorbed and released from the MOF layer and produce changes in the MOF layer’s refractive index near the nanohole array. These adsorption and desorption processes introduce shifts in resonant wavelength and intensity variations of the LSPR that a spectrometer can monitor. Likewise, these refractive index changes can be quantitatively analyzed through hue, saturation, and value (HSV) pixel depths from NHA images captured by a CMOS imaging device.

## 3. Materials and Methods

### 3.1. Device Fabrication

**Figure 1:**
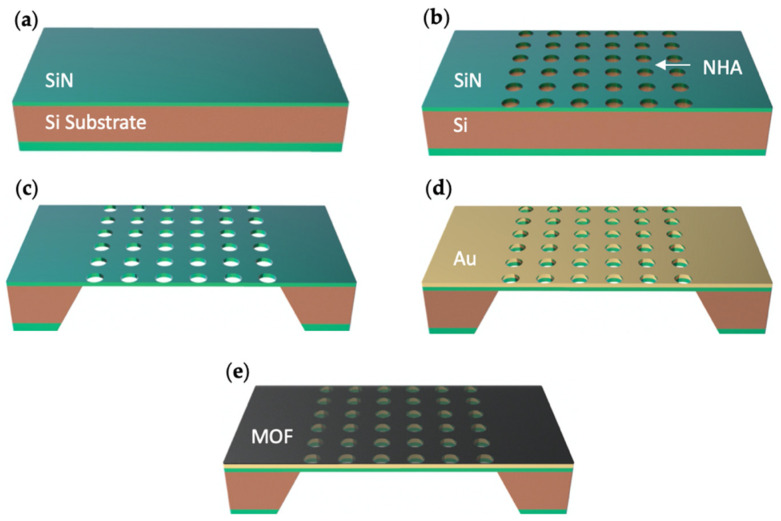
Schematic of nanohole array fabrication. Fabrication steps include: (**a**) LPCVD depositing silicon nitride layer; (**b**) EBL and RIE patterning NHA structures; (**c**) Mask aligner and RIE patterning membrane window; KOH wet etching silicon to create silicon nitride membrane; (**d**) E-beam evaporator depositing Ti/Au layer; (**e**) Coating Cu-BTC MOF layer.

The fabrication process for the NHA platform began with the deposition of 100 nm-thick silicon nitride on both sides of a 500 μm-thick silicon substrate via low-pressure chemical vapor deposition (LPCVD), as shown in Figure 1. The chip was then patterned with the NHA structure using e-beam lithography (EBL) and reactive ion etching (RIE). Next, a membrane window was patterned on the backside of the SiN layer with a mask aligner and RIE. KOH then etched the Si substrate to create the membrane, and 5 nm Ti and 75 nm Au were deposited on the Si/SiN using an e-beam evaporator.

After the bare NHAs structure was fabricated, the chip was then fully immersed in a 0.1 mmol/L 4-mercaptobenzoic acid (MBA, 99%, Sigma Aldrich [24], St. Louis, MO, USA)/ethanolic solution for 1 h to form a self-assembled monolayer. Next, the chip was fully immersed in a 1 mmol/L benzene-1,3,5-tricarboxylate (1,3,5-BTC, 98%, Acros Organics [24], Geel, Belgium)/ethanolic solution for 5 min, and then rinsed in ethyl alcohol (ethanol, 99.45%, Sigma Aldrich [24]) for 1 min. After that, the chip was fully immersed in a 1 mmol/L copper (II) acetate monohydrate (CuAc, 99%, Sigma Aldrich [24])/ethanolic solution for 5 min and then rinsed in ethanol for 1 min. One cycle of immersing the chip in 1,3,5-BTC solution and CuAc solution can form 1 layer (about 1 nm) of Cu-BTC MOF layer. The cycle was repeated 15 times to form a ≈15 nm-thick Cu-BTC MOF layer. The chip was dried in air for about 10 s in the transfer time between different solutions.

**Figure 2:**
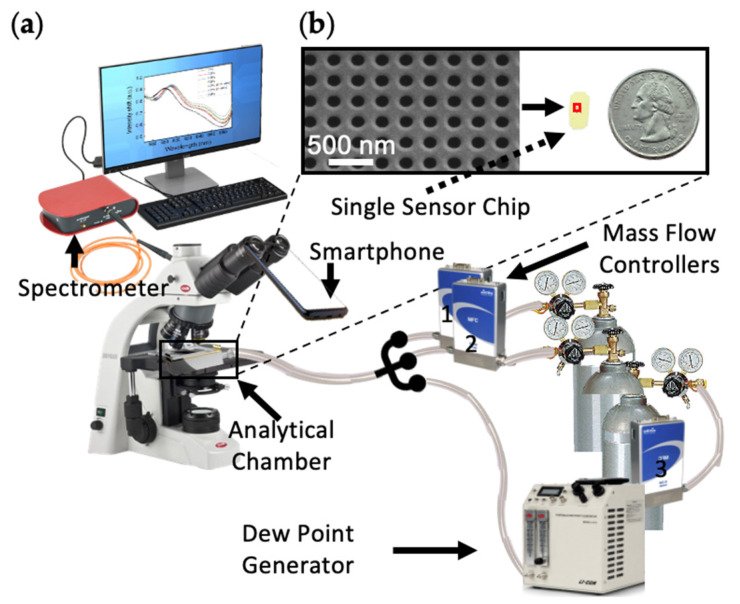
Sensing setup. (**a**) Diagram of the sensing system consisting of a spectrometer, a smartphone, microscope, NHA sensors, MFCs, and dew point generator. (**b**) SEM image of the bare NHA structure and an image of a single sensor chip versus a quarter. The chip had 4 NHA sectors, each with an area of 300 µm × 300 µm.

After fabrication, sensor chips were placed in a sealed analytical chamber, as shown in Figure 2a. Each sensor chip contained 4 NHA sectors (Figure S1a in Supplementary Material). An optical microscope image of a single NHA sector is shown in Figure S1b. Figure 2b shows a scanning electron microscope (SEM) image of part of the uncoated NHA structure (see also Figure S1c in Supplementary Material) with diameter and period of the nanoholes equal to 200 nm and 400 nm, respectively. More detailed process steps and characterization of the Cu-BTC MOF layer are provided elsewhere [18,20].

### 3.2. System Setup

The measurement setup was composed of a spectrometer (Thorlabs CCS 175 [24]), an optical fiber (Ocean Insight P600-1-VIS-NIR [24]), a microscope (Olympus BX41TF [24]), a light source (Olympus TH4-100 [24]), a sealed analytical chamber, NHA sensors, a gas-mixing manifold, a multi-gas controller (MKS 647c [24]), and a dew point generator (LI-COR LI-610 [24]), as shown in Figure 2a. An optical fiber, which transmits most efficiently from the visible to the near-infrared range, was used to connect the spectrometer to the microscope, equipped with 50× (NA = 0.45) and 20× (NA = 0.46) objective lenses. A CMOS imaging camera (embedded within an iPhone Xs [24]) can also be fixed onto the microscope with the camera aimed at one of the ocular lenses. Then, a sealed analytical chamber containing an NHA sensor was positioned on the stage of the microscope. The chamber was connected to the gas-mixing manifold (gas cylinders with tubing and mass flow controllers (MFCs, MKS [24]) to regulate the mixture of gas-phase components, including the background zero air, specified humidity level, and the target analytes). Humidity was controlled using a dew point generator.

As can be seen in Figure 2, three MFCs were connected to the sensing system, where MFC1 controlled the target analyte (acetaldehyde), MFC2 controlled the dry zero air (produced by passing air through a drier and a zero-air generator (Whatman [24])), and MFC3 controlled the moist air through a dew point generator (Table 1). The dew point generator was set to 21 °C to maintain the relative humidity (RH) of airflow controlled (upstream) by MFC3 to 90% RH at room temperature (23.1 °C). The flow rate of the gas mixture to the analytical chamber was 500 standard cubic centimeters per minute (sccm), which consisted of dry gas from MFC1 and MFC2 and humidified air from MFC3. Taking a 72% RH condition as an example, the flow rate of dry air from MFC2 is 100 sccm, and the humidified air (90% RH) from MFC3 is 400 sccm, which results in 72% RH air in an analytical chamber. Meanwhile, the target gas cylinder used here contained 0.001% (10 µmol/mol) acetaldehyde. When the flow rate from acetaldehyde cylinder was 50 sccm, the sensing platform was exposed to 1 µmol/mol acetaldehyde at a total flow rate of 500 sccm.

Blank experiments were run to confirm that the sensor responses to changes in humidity and acetaldehyde concentrations were not confounded by experimental artifacts. At high RH, an extra MFC (MFC4 as shown in Table 1) was added to examine the response of the sensor to blank exposures. Using an MFC matched to the analyte MFC, dry zero-air was added to the gas mixture rather than the 10 µmol/mol acetaldehyde source. The results of blank control experiments are shown in Figure S2 in Supplementary Material. The results shown in Figure S2 indicate that the intensity shifts caused by systematic error were about 10-times smaller than the intensity shifts caused by adding acetaldehyde to the gas mixture.

### 3.3. Experimental Measurements and Analysis Methods

Before the sensing measurements, a spectrometer calibration was performed to remove the background signal caused by the Au surface and the broadband light source. Spectrometer measurements were performed by sequentially exposing NHA sensors to different acetaldehyde concentrations, from 250 nmol/mol to 1000 nmol/mol (ppb), at room temperature. To explore the impact of RH levels, the RH was swept from 0% to 72% at 23.1 °C using a dew point generator. All RH levels are reported at lab ambient temperature (23 °C). The same experimental conditions were set and likewise monitored with a CMOS imaging device (smartphone). To quantitatively analyze the camera imaging results, NHA images were collected and processed with OpenCV, an open-source image processing package [24,25]. During the image processing, edges and centers of NHAs were first recognized. Then, the NHA areas were extracted from the surrounding background and aligned to the same angle based on their edges. Next, an algorithm in the OpenCV package was used to extract the HSV values for all the pixels (1600 × 1600) in each image, and the average values were calculated for further analysis. The extracted HSV datasets were presented in 3D scatterplots, and projections of these datasets on the HS-plane and corresponding trend curves were plotted and analyzed. Ellipses that covered each dataset projected on the HS-plane were generated with 95% confidence.

Principal component analysis (PCA), which allows one to better interpret datasets in a multidimensional coordinate system through reducing dataset dimensionality while preserving a maximum of original data information, was implemented to analyze the OpenCV-processed camera imaging results. H, S, and V values were set as variables, and NHA responses were set as samples in the PCA model. To better approximate the original datasets and minimize information loss, at least 95% of total variation needs to be explained by the principal components.

The repeatability of each experimental measurement was examined by calculating ±1 standard deviation of results captured from 3 to 5 different test trials on 2 to 4 sensor chips. Different testing trials represent measurements at different times. In each testing trial, 2 sensor chips were utilized for measurements, and 2 of the 4 NHA sectors on each sensor chip were randomly selected to perform the measurements in fixed experimental conditions. In total, 10 sensor chips were prepared in 4 batches (2 to 4 sensors for each batch) within a 1-year interval. Both spectrometer measurement and camera imaging results were processed and analyzed utilizing OriginPro [24].

## 4. Results and Discussion

### 4.1. Spectrometer Measurements

**Figure 3:**
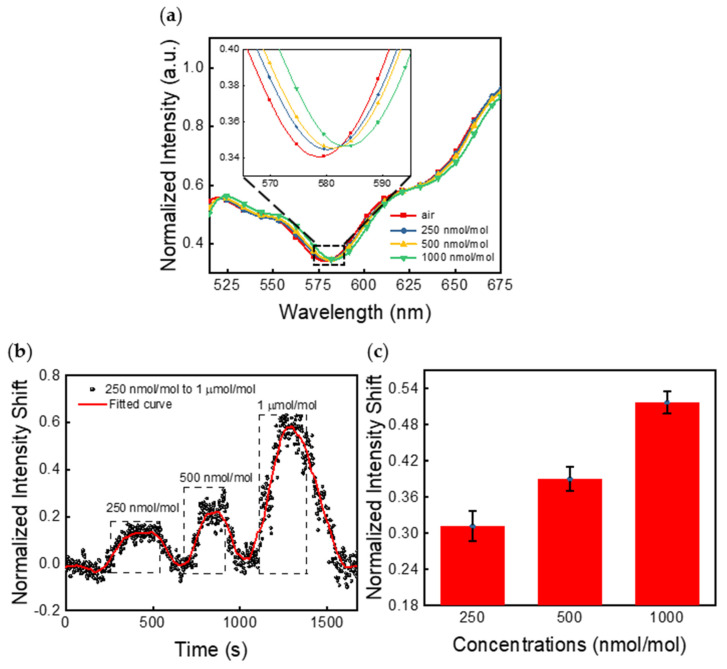
(**a**) Peak wavelength and intensity shift response of the NHA sensor when exposed to varying acetaldehyde concentrations in a dry air background. (**b**) The NHA sensor response to acetaldehyde for 250 nmol/mol, 500 nmol/mol, and 1 µmol/mol at a wavelength of 583.5 nm. The dotted boxes represent the input periods of the target analyte at each concentration. To help with visualization, a trend curve generated with the Savitzky–Golay method was overlaid. (**c**) Normalized intensity shifts of NHA sensors when exposed to varying acetaldehyde concentrations. Error bars along the *y*-axis represent ±1 standard deviation for measurement results captured in 5 different testing trials on 4 sensor chips.

**Figure 4:**
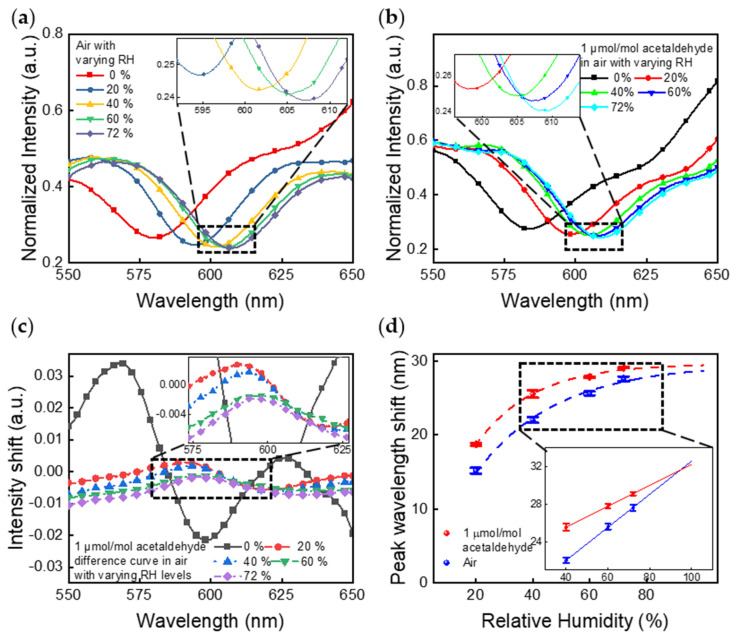
Peak wavelength and intensity shifts of the NHA sensor while the RH varies from 0% to 72% with (**a**) 0 µmol/mol acetaldehyde and (**b**) 1 µmol/mol acetaldehyde. (**c**) Responses, shown as difference curves, were obtained by subtracting from the response spectrum of the NHA to 1 µmol/mol of acetaldehyde in air (at each RH level), the response spectrum of 0 µmol/mol of acetaldehyde in air at the corresponding RH levels. (**d**) Summary of peak wavelength shifts vs. RH levels of each condition in (**a**,**b**). For high RH levels (≥40%), the sensor shows linear responses vs. RH levels. The estimated upper RH limit for detecting 1 µmol/mol acetaldehyde is about 95% RH based on the linear curve fitting with 95% confidence interval. The error bars represent ±1 standard deviation for measurement results captured from 4 different testing trials on 2 sensors.

**Figure 5:**
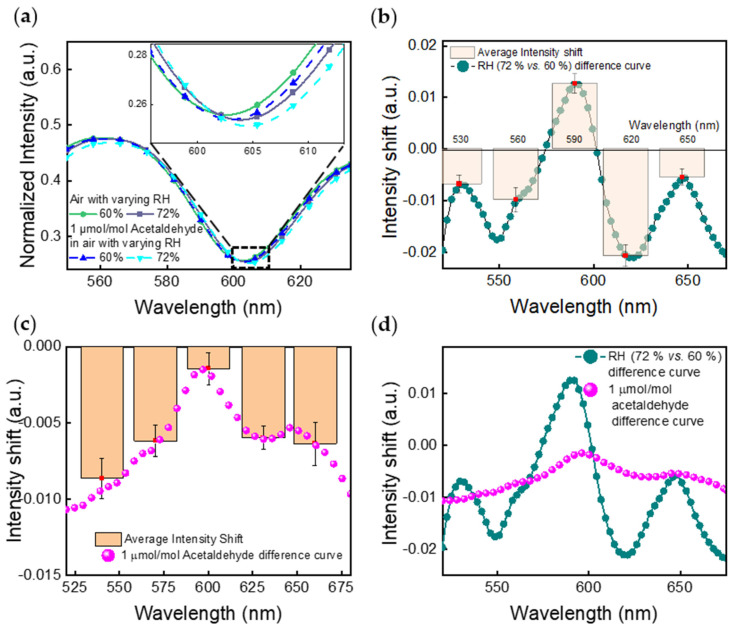
(**a**) Peak wavelength and intensity shifts of the NHA when exposed to air with 0 µmol/mol acetaldehyde and with 1 µmol/mol of acetaldehyde, both in backgrounds with 60% RH and 72% RH. (**b**) Response, shown as a difference curve, was obtained by subtracting the spectrum of the NHA in 60% RH air from the spectrum in 72% RH air. (**c**) Response, shown as a difference curve, was obtained by subtracting the spectrum of the NHA in 0 µmol/mol of acetaldehyde in air with 72% RH from the spectrum in 1 µmol/mol of acetaldehyde in air with 72% RH. Error bars along the *y*-axis represent ±1 standard deviation for measurement results captured in 3 different testing trials from 4 sensor chips. (**d**) Comparison of the difference curves generated for (**b**,**c**).

The spectrometer-measured results are presented in Figure 3, Figure 4 and Figure 5. Figure 3 shows the measured NHA responses when exposed to varying concentrations of acetaldehyde, from 250 nmol/mol to 1 µmol/mol, in a dry air background. As shown in Figure 3a, both the peak wavelength and normalized intensity shifted, even for the lowest acetaldehyde concentration. Figure 3b shows data for the continuous measurements of acetaldehyde. The response curve follows dosing at three concentrations, and the response feature in each case has two parts, an adsorption part (rising) and a desorption part (falling).

Figure 3c summarizes the changes in the normalized intensity upon exposure to acetaldehyde, with error bars representing ±1 standard deviation for 100 measurements at each concentration: 250 nmol/mol, 500 nmol/mol, and 1000 nmol/mol. To examine the repeatability, the average intensity shift and the error bar at each concentration were calculated from these datapoints that were measured in five different testing trials on four sensor chips. These datapoints for each concentration were all captured from saturation regions (the flat curve region in each dotted box as shown in Figure 3b). In total, 20 curves similar to the ones in Figure 3b were measured, and 5 datapoints were randomly collected from saturation regions in each curve.

Figure 4 presents the sensing performance in air with varying RH levels measured by a spectrometer. As shown in Figure 4a,b, the NHA sensors underwent shifts of peak wavelength and normalized intensity when the RH level was varied from 0% to 72% with 0 µmol/mol acetaldehyde and 1 µmol/mol acetaldehyde, respectively. The NHA responses of 20% to 72% RH without and with 1 µmol/mol acetaldehyde were generated as difference spectra shown in Figure S3a,b in Supplementary Material, respectively. The spectral changes caused by humidity were significant.

Nevertheless, when the NHA sensors are exposed to a background with RH levels varying from 20% to 72% at 23.1 °C, the spectrometer can still recognize clear differences in the peak wavelength and intensity between air with and without 1 µmol/mol acetaldehyde, as illustrated in Figure 4c,d and Figure 5a. Figure 4c presents NHA responses, shown as difference curves, which were obtained by subtracting from the response spectrum of the NHA to 1 µmol/mol of acetaldehyde in air with each RH level to the response spectrum of 0 µmol/mol of acetaldehyde in air at the corresponding RH levels. As the RH level increased, these difference curves gradually became indistinguishable. In Figure 4d, the NHA responses caused by 1 µmol/mol acetaldehyde in each RH level are presented as peak wavelength shifts versus RH levels. For high RH levels (≥40%), the sensor shows linear responses vs. RH levels. The estimated upper RH limit for detecting 1 µmol/mol acetaldehyde is about 95% RH based on the linear curve fitting with a 95% confidence.

To further compare spectrum shifts caused by RH changes and by acetaldehyde concentration changes (from 0 µmol/mol to 1 µmol/mol) at high RH levels, Figure 5a shows response spectra of the NHA when exposed to air with 60% RH, air with 72% RH, and air plus 1 µmol/mol acetaldehyde with 60% RH and 72% RH at 23.1 °C. Figure 5b,c show the corresponding shift of intensity versus wavelength through the subtraction between the spectra from sensors exposed to two groups of conditions: (I) Air with 72% RH and 60% RH; (II) 1 µmol/mol acetaldehyde and 0 µmol/mol acetaldehyde, both in a background of air with 72% RH at 23.1 °C, respectively. Figure 5d directly compares the difference curves generated for Figure 5b,c (difference curve for air with 60% RH is presented in Figure S4 in Supplementary Material). One can notice different intensities, peak locations, and numbers of peaks in those two difference curves representing changes in humidity or acetaldehyde. The repeatability of the spectral changes was examined by calculating ±1 standard deviation of the intensity shift at five uniformly distributed wavelength positions with an interval of 30 nm along the entire spectrum. The 48 results were captured from 8 randomly selected NHAs (from 4 different sensor chips) in 3 different testing trials with fixed experimental setups. These four sensors were prepared in two batches (two sensors for each batch) within a 2-month interval.

### 4.2. Camera Imaging

**Figure 6:**
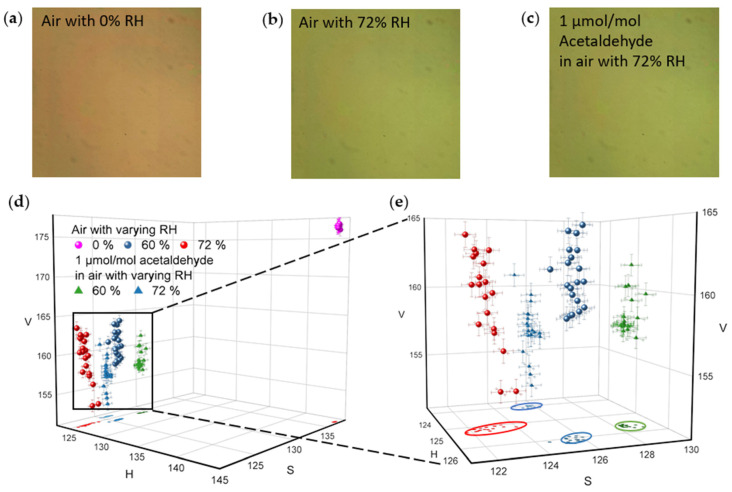
NHA color variation. (**a**–**c**) show raw images of a single NHA when exposed to air with 0% RH, air with 72% RH, and 1 µmol/mol of acetaldehyde in air with 72% RH, respectively. All images were captured by a CMOS imaging device. (**d**,**e**) HSV plot of the NHA responses to air with varying RH levels (0%, 60%, and 72%) and to 1 µmol/mol of acetaldehyde in air with varying RH levels (60% and 72%). Error bars along the H-axis, S-axis, and V-axis represent ±1 standard deviation for H, S, and V values of each pixel in each image, respectively. Ellipses on the HS-plane that cover each dataset were generated with 95% confidence.

**Figure 7:**
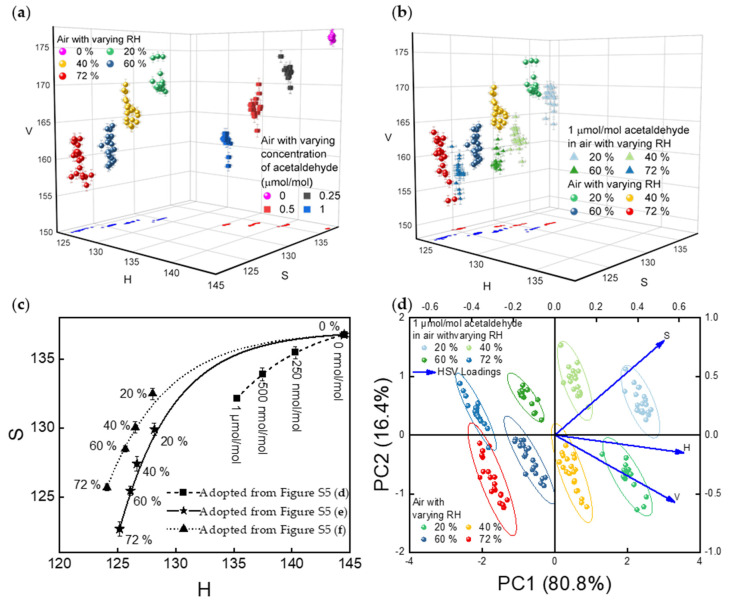
(**a**) Comparing NHA sensors’ responses to varying concentrations of acetaldehyde (0 to 1 µmol/mol) and varying RH levels (0%, 20% 40%, 60%, and 72%). (**b**) Comparison of NHA sensors’ responses to varying RH levels with and without 1 µmol/mol acetaldehyde. (**c**) Comparison of trend curves in Figure S5d–f. The error bars along the *x*-axis and *y*-axis represent ±1 standard deviation of H and S for data collected from 4 different testing trials on 2 sensor chips, respectively. Please note that for relatively small error values, the data labels may obscure the error bars. (**d**) PCA result of NHA sensors’ responses to varying RH levels with and without 1 µmol/mol acetaldehyde. The first 2 PCs account for 97.2% (≥95%) of the total variation. Ellipses that cover each dataset were generated with 95% confidence.

Figure 6 and Figure 7 show sensing performance results of NHA sensors measured by a CMOS imaging device under air with varying acetaldehyde concentrations and varying RH levels. Figure 6a–c show a group of raw CMOS images of a single NHA when the sensor is exposed to air with 0% RH, air with 72% RH, and air plus 1 µmol/mol acetaldehyde with 72% RH at 23.1 °C, respectively. Similar to the significant spectral shift shown in Figure 4a and Figure 6a,b have a visible color change caused by the impact of humidity, whereas the color changes are not easily visible between Figure 6b,c, which matches the small spectral shifts present in Figure 5a. The color changes interpreted by visual inspection are highly subjective. Hence, to further quantify the color changes in NHA’s images caused by the target gas and the humidity, images were collected and processed with OpenCV package.

Figure 6d,e show 3D scatterplots for the extracted HSV datasets. The datasets were acquired by processing of 100 images obtained from the sensor exposed to 5 conditions (20 images from each condition): (I) Dry air, (II) air with 60% RH, (III) 1 µmol/mol acetaldehyde in the air with 60% RH, (IV) air with 72% RH, and (V) 1 µmol/mol acetaldehyde in the air with 72% RH, all at 23.1 °C. The differences between dry and humid air samples in Figure 6d are apparent. However, Figure 6e indicates there is also a discernible difference among the conditions (II), (III), (IV), and (V). Datasets of these four conditions were also projected on HS-plane and covered by ellipses with 95% confidence. On the HS-plane, the difference among these four conditions is further distinguishable, as shown by the ellipses in Figure 6e. Next, NHAs sensor responses were further analyzed in Figure 7.

Figure 7 shows the results from the analysis of camera images obtained under similar experimental conditions to the spectra discussed above (as well as Figure S5 in the Supplementary Material). Responses of NHAs, when exposed separately to varying concentrations of acetaldehyde from 250 nmol/mol to 1 µmol/mol in dry air and to different RH levels, are presented in Figure 7a (and Figure S5a). In Figure 7b (and Figure S5b,c), the average HSV values of the camera images are shown for responses to the presence of 1 µmol/mol acetaldehyde in air with varying RH levels. The single-variable datasets plotted in Figure S5a–c are projected onto HS-plane to further demonstrate the response difference, as shown in Figure S5d–f, respectively. The response differences between varying concentrations, and RH levels are distinguishable on HS-plane projections. The comparison of NHA sensor responses to (I) varying concentrations of acetaldehyde and varying RH levels and (II) varying RH levels with and without 1 µmol/mol acetaldehyde are also plotted in Figure 7a,b, respectively. Trend curves were drawn for these datasets and are summarized in Figure 7c, where the curves represent the changes in NHA responses to varying conditions. Note that the HS values in a zero-air background are quite distinct from the HS values in the presence of humidity (with or without acetaldehyde). This outcome agrees with the spectrometer results shown in Figure 4 and visual inspection results shown in Figure 6.

PCA methods were also applied to further discriminate between the NHA responses plotted in Figure 7b. As shown in the biplot of Figure 7d, principal component (PC) 1 and PC 2 account for 80.8% and 16.4% of the total variation around PCs, respectively. Together, PC1 and PC2 account for 97.2% (≥95%) of the total variation, which means the 2D scatterplot shown in Figure 7d provides a good approximation to the original datasets that were presented in the 3D scatterplot shown in Figure 7b. One can infer that the datasets move along the *x*-axis as the RH level changes and move along the *y*-axis when the concentration of acetaldehyde changes (from 0 µmol/mol to 1 µmol/mol). Figure 7d also reveals a clear separation between the NHA responses to varying RH levels with and without exposure to 1 µmol/mol acetaldehyde. That is, the PCA plot shows that the sensor responses to relative humidity and to acetaldehyde are distinct and distinguishable. Ellipses that cover each dataset were generated with 95% confidence. These datasets were acquired by processing NHA images that were captured in four different testing trials on two sensors. These outcomes measured by a CMOS imaging device are consistent with the results measured by a spectrometer. Thus, a smartphone embedded CMOS camera can provide discrimination capability that is comparable to a spectrometer.

Compared to a spectrometer, utilizing a CMOS camera for testing consumes less data acquisition time (by about 120×) and costs less (by about 70×). In addition, considering the potential advantage of applying ANN algorithms for image processing to assist the analysis of the measurements further, the camera approach is believed to be a promising improvement to enhance the performance of an integrated plasmonic sensing platform.

## 5. Conclusions and Perspectives

We have developed the initial stage of a technology that shows potential as a gas-phase breath analysis tool for monitoring a biomarker (acetaldehyde) related to CF. The paper presents a plasmonic sensing platform, which has, for the first time, successfully detected acetaldehyde at nmol/mol (ppb) levels. The platform, which is based on LSPR and utilizes a MOF coating, can also differentiate between 0 µmmol/mol acetaldehyde and 1 µmol/mol acetaldehyde in a background of air with as high as 72% RH, both with a spectrometer and with a CMOS imaging device (smartphone camera). The results for these two methods were similar in terms of their monitoring and discrimination capabilities but we have described certain cost and performance advantages that are possible for camera-based data acquisition.

We note that, for practical development of a breath-based sensor, RH in exhaled breath is over 90%. The high RH impacts the sensing performance, which requires further improvement for breath sample measurements containing water vapor. Further challenges are found with the complexity of exhaled breath, which has a large number of unique compounds, including other VOCs. As mentioned in the Introduction, multiple CF biomarkers exist in exhaled breath, such as ethanol, methanol, and 2,3 butanediol. Monitoring these targets with the proposed platform would offer additional analytical information. Other approaches to address these complications will also be investigated in future work. One approach would be to employ the demonstrated platform as a detector with a GC front end. Another approach would aim to develop a fully miniature system with higher selectivity. To this end, we are continuing to examine the capabilities of the technology to deal with discriminating and tracking multiple biomarkers by utilizing varied surface/interfacial functionalization, features at multiple wavelengths and temperature variations [18]. Incorporating such methods and specifically designed image analysis algorithms with a camera-based system could result in an inexpensive and highly capable POC device.

## Figures and Tables

**Table 1:** MFCs controlled gas varieties, ranges, and accuracy.

MFC	Gas Varieties	Control Ranges ^1^ (sccm)
MFC1	acetaldehyde	1 to 50
MFC2	dry air	20 to 2000
MFC3	moist air (90% RH)	20 to 2000
MFC4 ^2^	dry air	1 to 50

^1^ Typical accuracy: ±1% of full scale. ^2^ MFC4 was used for blank tests.

## Data Availability

Not applicable.

## Supplementary Materials

The following are available online at https://www.mdpi.com/article/10.3390/s21113776/s1, Figure S1: Schematic of nanohole array sensor chip. Figure S2: Blank control experiment results in air with 72% RH. Figure S3: Difference curves of varying RH levels. Figure S4: 1 µmol/mol of acetaldehyde difference curve in air with 60% RH. Figure S5: HSV 3D plot of NHA sensors response when exposed to varying acetaldehyde concentrations, varying RH levels, and 1 µmol/mol acetaldehyde in air with varying RH levels.
