# Supplementary material for: Plasmonic Sensing Studies of a Gas-Phase Cystic Fibrosis Marker in Moisture Laden Air
Source: Sensors (Basel). 2021 May 29;21(11):3776. doi: 10.3390/s21113776 (PMC8197828; doi:10.3390/s21113776)
Supplement: Supplementary file 1 [file sensors-21-03776-s001.zip › sensors-1212240-SI.pdf]

sensors

Supplementary Materials

Plasmonic Sensing Studies of a Gas-Phase Cystic Fibrosis Marker in Moisture Laden Air

Libin Sun [1,2], Douglas Conrad [3], Drew A. Hall [4], Kurt D. Benkstein [2], Steve Semancik [2], and Mona E. Zaghloul [1,*]

1 School of Engineering and Applied Science, George Washington University, Washington, DC 20052, USA
2 Biomolecular Measurement Division, National Institute of Standards and Technology, Gaithersburg, MD 20899, USA; kurt.benkstein@nist.gov (K.D.B.); stephen.semancik@nist.gov (S.S.)
3 Department of Medicine, University of California, San Diego, La Jolla CA 92037, USA; dconrad@health.ucsd.edu
4 Department of Electrical and Computer Engineering, Jacobs School ofEngineering, University of California, San Diego, La Jolla, CA 92093, USA; drewhall@ucsd.edu
* Correspondence: libinsun@gwu.edu (L.S.); zaghloul@gwu.edu (M.E.Z.)

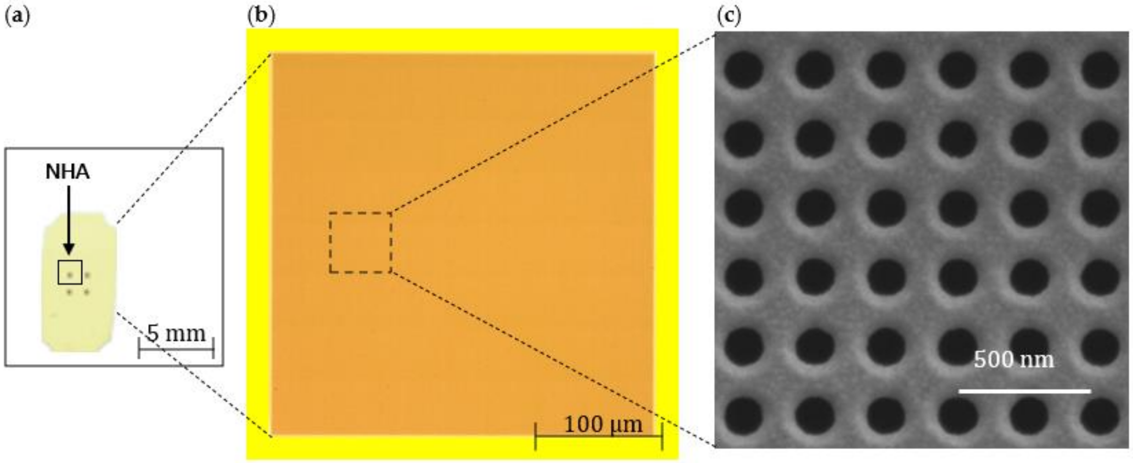

Figure S1. Schematic of a nanohole array sensor chip. (**a**) A single sensor chip with four NHA sectors, each with an area of 300 µm × 300 µm. (**b**) Optical microscope image of a single NHA sector. (**c**) SEM image of part of NHA structure.

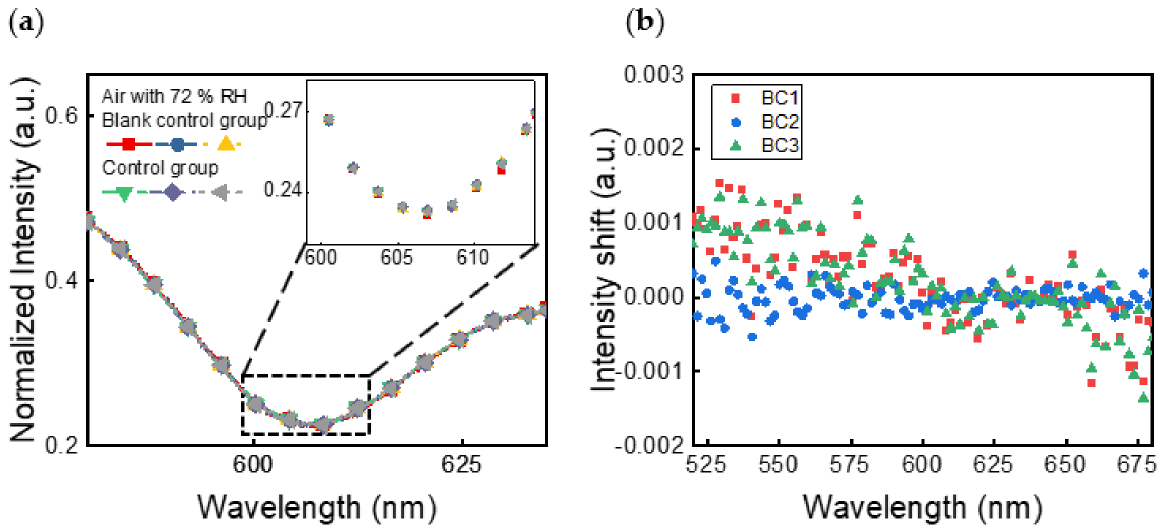

Figure S2. Blank control experiment results in air with 72 % RH. (**a**) Response spectra of the blank control group and the control group. (**b**) Responses, shown as difference curves obtained by subtracting response spectra of the blank control

group from the control group. In the blank control group, MFC2 was set to output 100 sccm dry air, and MFC3 was set to output 400 sccm moist air with 90 % RH. In the control group, both MFC2 and MFC4 were set to output 50 sccm dry air, and MFC3 was set to output 400 sccm moist air with 90 % RH. 'BC' denotes difference curve of the blank control experiment.

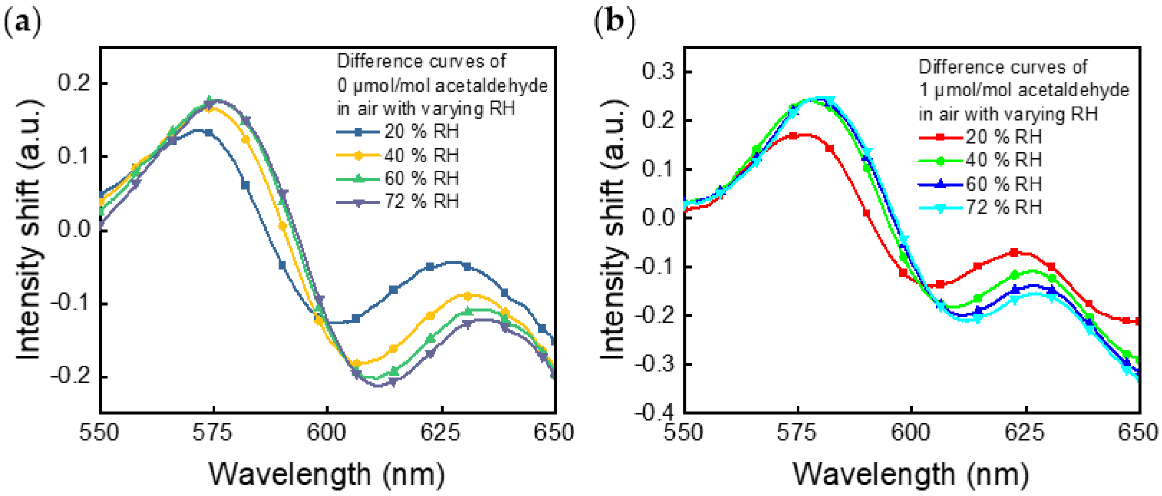

Figure S3. Difference curves of varying RH levels. Responses are shown as difference curves of the NHA sensors to varying RH levels with (**a**) 0 μmol/mol acetaldehyde and (**b**) 1 μmol/mol acetaldehyde. The response spectra from the sensor in dry air (with and without 1 μmol/mol acetaldehyde) were subtracted from each of the spectra collected at the other RH levels (with and without 1 μmol/mol acetaldehyde).

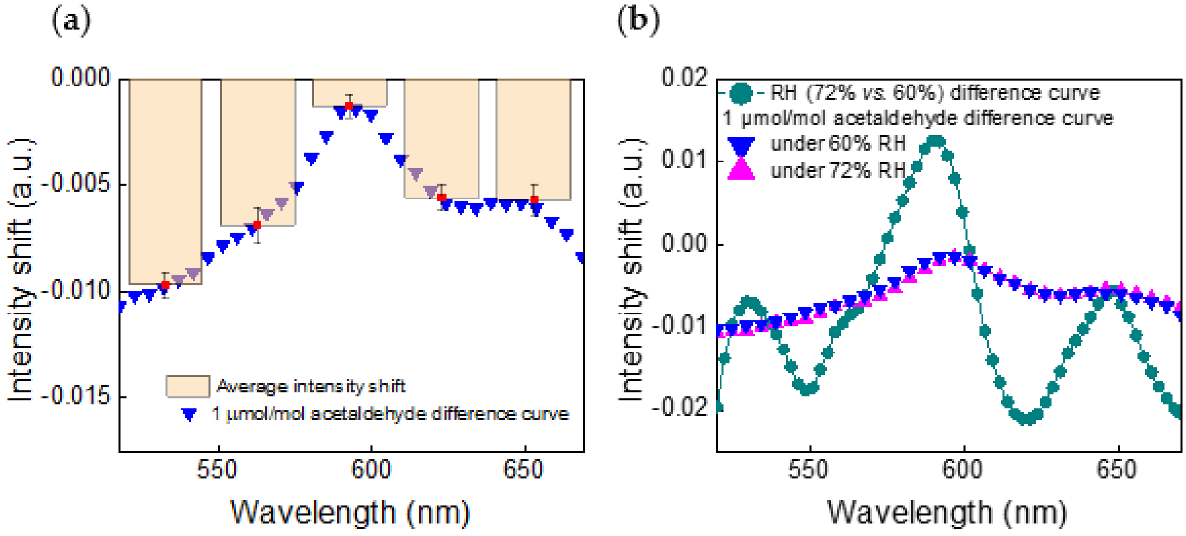

Figure S4. 1 μmol/mol of acetaldehyde difference curve in air with 60 % RH. (**a**) Response, shown as a difference curve, obtained by subtracting the spectrum of the NHA with 0 μmol/mol of acetaldehyde in air with 60 % RH from 1 μmol/mol of acetaldehyde in air with 60 % RH. Error bars along the y-axis represent ± one standard deviation for measurement results captured in three different testing trials from four sensor chips. (**b**) Comparison of the difference curves generated for (**a**) and Figure 5(**d**).

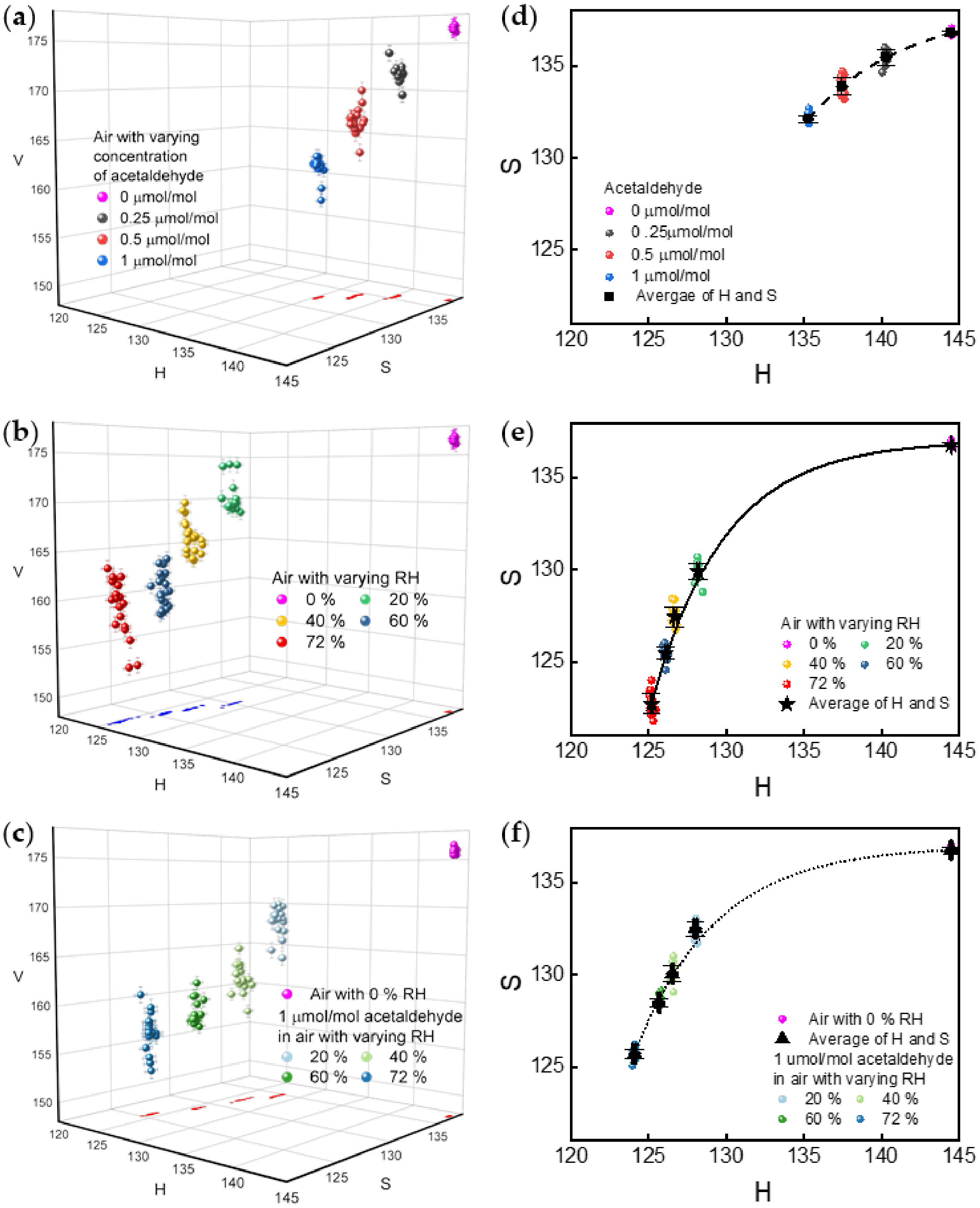

Figure S5. HSV 3D plot of NHA sensors response when exposed to (**a**) varying acetaldehyde concentrations from 0 nmol/mol to 1 µmol/mol; (**b**) varying RH levels from 20 % to 72 %; (**c**) 1 µmol/mol acetaldehyde in air with varying RH levels from 20 % to 72 %. (**d**), (**e**) & (**f**) Projections of data sets plotted in (**a**), (**b**) & (**c**) on HS-plane, respectively. Trend curves were drawn for data sets in (**d**), (**e**) & (**f**) with 95 % confidence, respectively. The error bars along the x-axis and y-axis represent ± one standard deviations of H and S for data collected from four different testing trials on two sensor chips, respectively. Please note that for relatively small error values, the data labels may obscure the error bars.
